# Comparative Evaluation of Efficacy and Safety of the Diode Laser (980 nm) and Sclerotherapy in the Treatment of Oral Vascular Malformations

**DOI:** 10.1155/2022/2785859

**Published:** 2022-09-05

**Authors:** Peeyush Shivhare, Naqoosh Haidry, Neha Sah, Ajay Kumar, Abhishek Gupta, Ankur Singh, Mohan Raju Penumatcha, Shalini Subramanyam

**Affiliations:** ^1^Department of Dentistry, All India Institute of Medical Sciences, Patna 801507, India; ^2^Department of Oral and Maxillofacial Surgery, Dental College Azamgarh, Azamgarh 276128, India; ^3^Department of Oral Medicine and Radiology, Faculty of Dental Sciences, Institute of Medical Sciences, Banaras Hindu University, Varanasi 221005, India; ^4^Department of Oral Medicine and Radiology, Chitwan Medical College, Bharatpur, Chitwan 44207, Nepal; ^5^Department of Oral Medicine and Radiology, Narsinhbhai Patel Dental College and Hospital, Visnagar 384315, India; ^6^Private Dental Clinic, Secunderabad 500003, India; ^7^Private Dental Clinic, Bangalore 560038, India

## Abstract

**Background:**

Vascular malformations are structural abnormalities which are formed by progressively enlarging aberrant and ecstatic vessels without endothelial cell proliferation and composed of the type of vessel involved, i.e., capillary, veins, and arteriovenous. Treatment of vascular malformations may involve many techniques like sclerotherapy, embolization, surgical resection, cryotherapy, laser treatment, or medical therapy. This observational prospective study is aimed at evaluating and comparing the effects and efficacy of diode laser and sclerotherapy in the treatment of oral vascular malformation.

**Materials and Methods:**

40 patients presenting with oral vascular malformation were included in the present study. The patients were divided equally (20 in each) into two groups, i.e., the laser group and sclerotherapy group. Sclerotherapy was performed with 3% sodium tetradecyl sulfate while the laser group was treated with diode laser 980 nm with transmucosal thermophotocoagulation. The patients were assessed for the response, remission, and side effects. The results obtained were tabulated and compared with the chi-square test.

**Results:**

Side effects were found significantly lesser in the laser group compared to the sclerotherapy group (*p* < 0.05). Statistically significant difference was seen for postoperative pain between two groups. The laser group had mild to moderate pain compared to severe pain in the sclerotherapy group. Recurrence was observed more in the laser group compared to the sclerotherapy group.

**Conclusions:**

Laser and sclerotherapy with 3% sodium tetradecyl sulfate both are effective in the treatment of vascular malformations. Diode laser seems to be better than sclerotherapy given lesser side effects and comfort to the patients while sclerotherapy seems to be better in respect to recurrences.

## 1. Introduction

Vascular lesions or anomalies can occur anywhere in the body and form a heterogeneous group of congenital lesions of vascular origin. The first classification of the vascular lesion was given by Mulliken and Glowacki in [1] based on endothelial characteristics, the classification was accepted by the International Society for the Study of Vascular Anomalies (ISSVA) 1996 [2]. Based on the natural history, cellular turnover, and histology, vascular lesions are classified into vascular tumors such as hemangioma and vascular malformations [2]. ISSVA updated this system in 2014 and classifies vascular anomalies into proliferative vascular tumors and nonproliferative vascular malformations. Vascular tumors are further divided into benign, locally aggressive/borderline, and malignant, while vascular malformations are divided into simple, combined, vascular malformation of major vessels and malformation associated with other anomalies. This updated classification was approved by the General Assembly in Amsterdam, the Netherlands in May 2018 [3].

Hemangiomas are lesions which are usually an infantile (common) or congenital (rare) forms of benign tumor with vascular tissues that arises from the rapid proliferation of endothelial cell population. Hemangioma usually appears during infancy and usually has a history of proliferation (proliferative phase) followed by spontaneous involution (involution phase) [2, 4].

Vascular malformations (VM) are congenital lesions that occur frequently in the oral cavity and head and neck region. They are characteristically bluish in color, compressible in nature, and nonpulsatile on examination. These malformations become more visible with time. Sometimes, they are unable to be detected in the early stages of development. Based on the blood vessel that is associated with the lesion, the vascular malformations are further classified into (a) arterial, (b) arteriovenous, (c) venous, (d) capillary, and (e) lymphatic malformations [2, 4]. Treatment of vascular lesions may involve surgical resection, embolization, cryotherapy, medical therapy (cortisone, beta-blockers), sclerotherapy, or laser treatment [4, 5].

Laser stands for an acronym, “Light Amplification by Stimulated Emission of Radiation.” Through a process of optical amplification, a laser device releases electromagnetic radiation. Light usually follows a principle of “spontaneous emission” unlike laser machine follows another “stimulated emission.” Different laser systems have been used clinically in the treatment of hemangiomas, vascular malformations such as carbon dioxide (CO_2_) laser, argon, diode, erbium-doped yttrium aluminum garnet (Er:YAG), potassium-titanyl-phosphate (KTP), pulsed dye laser (PDL) and neodymium-doped yttrium aluminum garnet (ND:YAG) lasers [6]. These two different techniques for treating vascular lesion have been discussed in various literatures, i.e., (a) the *trans*-mucosal thermocoagulation (TMT) and (b) the intralesional photocoagulation (ILP). The TMT is attained when the laser irradiation is transmitted at a distance of 2-3 mm from the surface, without contact of the fiber with the tissue (noncontact mode). As it passes through the tissues, the laser beam coagulates the tissue by generating heat to a depth of around 7 to 10 mm by *photocoagulation* where processes like dehydration (removal of fluid) and whitening (change in color) of the hemangioma can be detected on simple observation instantaneously, during postoperative recovery. The intralesional photocoagulation is attained when the laser irradiation is transmitted by contacting the fiber tip with the tissue and the successive release of energy directly into the lesion. Therefore, perforating the lesion is encouraged, which also forces the withdrawal of its contents (usually blood). This specific technique is very useful for lesions located on the lip, tongue, cheek, and mucosa which are large in nature and deep inside [7, 8].

Sclerotherapy is a procedure that eliminates small vessels, varicose veins, and vascular anomalies by the method of injecting a biomaterial called *sclerosant*. A sclerosing agent causes discernible tissue irritation, with minimal thrombosis causing endothelial damage, and subsequent inflammation and tissue necrosis locally. The consequence of this inflammation and tissue necrosis is the fibrosis and contracture, which ultimately results in the disappearance of the lesion [9]. Based on their chemical properties and their mechanism of action, sclerosing agents are classified into the following: (a) detergents—it disrupts the vein cellular membrane, including sodium tetradecyl sulfate, polidocanol, sodium morrhuate, and ethanolamine oleate. (b) The osmotic agents impairs the cell by shifting the water balance, examples being hypertonic sodium chloride solution and sodium chloride solution with dextrose. (c) The sclerosants like chromated glycerin, polyiodinated iodine, OK 432, and bleomycin are the chemical irritants which are known to damage the cell wall. Bleomycin, polidocanol (POL), and sodium tetradecyl sulfate (STS) are the commonly used sclerosants due to their noted efficacy and safety profile [9, 10].

There is literature focusing on either the application of sclerotherapy or laser in the treatment of oral vascular malformation but no study is published contrasting these two. Thus, an observational prospective study is aimed at evaluating and comparing the effects and efficacy of two different treatment modalities for vascular lesion/vascular malformation, i.e., laser and sclerotherapy were designed and carried out.

## 2. Materials and Methods

This observational study was exclusively researched, coordinated, and conducted in the Department of Oral Medicine and Radiology, Nobel Medical College Teaching Hospital, Biratnagar, Nepal. Informed consent was obtained from all the study participants/patients along with an ethical clearance obtained from the institutional ethical committee board. All procedures performed in the study were conducted in accordance with the ethical standards provided by “World Medical Association Declaration of Helsinki on ethical principles for medical research involving humans for studies.” Ethics approval was obtained from the ethical review committee of the same institution with IRC reference no. IRC-NMCTH 317/2019 on 22/10/2019. The present research was done in line with the Strobe Statement (http://strobe-statement.org/) [11].

### 2.1. Study Population, Setting, and Design

This study included a total of 40 subjects, presenting with oral vascular malformations reported to the Department of Oral Medicine and Radiology, Nobel Medical College and Hospital. These subjects were divided equally into two groups (20 in each), i.e., the laser group and sclerotherapy group. The patients in both the groups were sampled based on the site and size of the oral vascular malformations by a purposive nonprobability sampling method.

### 2.2. Inclusion Criteria

Cases of low-flow vascular malformation confirmed with Doppler ultrasound and CT angiogram were included in the study.

### 2.3. Exclusion Criteria

Vascular malformations associated with systemic anomalies or extending beyond the oral cavity and high-flow vascular malformations were excluded from the study.

### 2.4. Methodology

**Figure 1:**
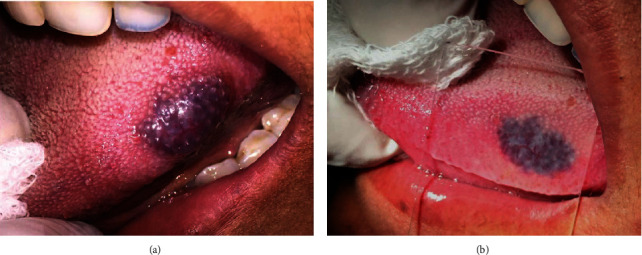
Diascopy test.

Meticulous medical history of each participant was taken along with the patient's age, gender, the site of the vascular lesion, its time of onset, and dimensions were all recorded. A diascopy test was performed chair side (Figure 1).

All patients were subjected to a Doppler ultrasonography and CT angiogram. Transmucosal thermophotocoagulation (TMT) was performed with a diode laser (980 nm, Indilase) for the laser group, and 3% sodium tetradecyl sulfate was used for sclerotherapy.

**Figure 2:**
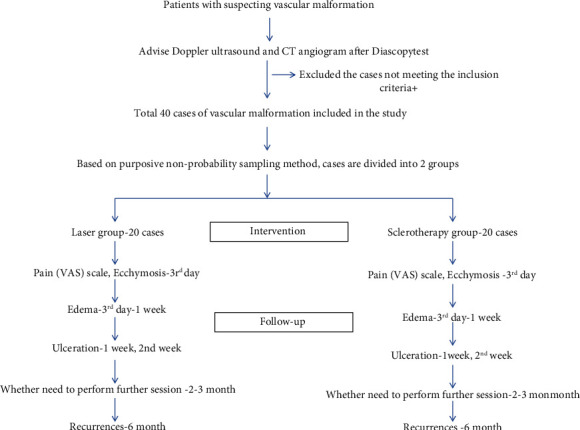
Flow chart to summarize the research protocol.

The following variables: age, gender, site of lesions, size of lesions, number of sessions, details of the side effects like pain (VAS scale on 3^rd^ postoperative day), edema, ulceration, and ecchymosis, the response of treatment to individual groups, and details of the recurrences were studied. Pain and ecchymosis were observed in 3^rd^ day postoperatively. A VAS of 100 mm in length will be used to evaluate the intensity of pain. This scale converted the visual analogical evaluation made by the patient into a numerical value: from 0 (corresponding to 0 mm on the VAS and showed no pain) to 10 (100 mm on the VAS and showed unbearable pain) [12]. Intraoral edema was evaluated by comparing the wound area with the anatomical area of the opposite side for presence or absence of asymmetry on 3^rd^ day and 1 week postoperatively [13]. Ulceration, ecchymosis, and recurrences were evaluated by the investigator based on his clinical observation. The patient was evaluated after 6 months for recurrences. The research protocol is summarized in Figure 2. These observations were reassessed by another investigator to avoid observer bias. Extraoral and intraoral photographs of the lesion's area were taken before and after the treatment(s) and at each of the follow-up assessments done periodically. The collected data were analyzed with IBM, SPSS statistics software version 24.0 (IBM INC, Illinois, USA). Chi-square testing was used for categorical data. A *p* < 0.05 was considered statistically significant.

### 2.5. The Procedure of Transmucosal Thermophotocoagulation (TMT) by Diode Laser (980 nm, Indilase, India) (Figure 3)

Perilesional infiltration of local anesthetic (2% lignocaine without epinephrine) was used. A diode laser (Ga-Al-As, DILAS, Germany) at 980 nm wavelength (model: IndiLase, MEDSOL, Hosur, India), operating in a continuous mode with an output power of 2.0–3.0 W and a flexible 400-mm diameter optic fiber (polyamide, 400 microfibers; Medfibers) equipped with a hand piece, was used to treat low-flow vascular malformations. The energy transmitted to the tissue was on average 1587.30 J/cm^2^ (Here, the diameter of optic fiber is 400 *μ*m/0.4 mm; thus, area will be *πr*^2^ = 0.126 mm^2^/0.0126 cm^2^. Energy = 2 *W*∗1 sec = 2 Joule. For 1 cm^2^ area, energy transfer is 1/0.00126 = 793.65∗2 J = 1587.30 J/cm^2^ (2380.95 J/cm^2^ for 3 watt). The duration of the treatment varies on the basis of clinical shrinkage and whitening of the lesion. Thus, total energy transfer depends on the size and treatment duration of the lesion. Complete aseptic conditions were maintained, and both the patient and operator were given protective eyeglasses to wear as protective measures.

The initiated laser fiber was kept about 2 mm away from the lesion's surface using a transmucosal coagulation method. A thin glass slide was used as a barrier to avoid accidental contact of the initiated tip to the lesion. Treatment was initiated at the lesion's peripheral edges moving towards the center with continuous inward rotatory movements, never remaining in the same place for long; the area being treated was irrigated with a cold physiological solution to avoid deep thermal injury. The lesion was irradiated till the clinical evidence of shrinkage and whitening appears; the laser beam was then switched off. This technique is called “forced dehydration,” and this effect is seen as hemoglobin in the blood which strongly absorbs the laser energy with a consequent generation of heat and tissue coagulation, extending approximately 7–10 mm deep [8, 14].

Compartmentalization and serial steps technique [15] were preferred for lesions more than 3.1 cm in order to avoid excessive thermal damage to surrounding tissues and better wound healing. The lesions were divided into multiple compartments. Each compartment was then treated in subsequent sessions based on the healing period, usually 3 months.

Analgesics were prescribed postoperatively only when required. A topical antiseptic solution (betadine 2%) was prescribed for oral rinse to all the patients to prevent secondary infection. They were also instructed to avoid eating hard textured foods or hot foods for the first 3 days postintervention. These patients were reevaluated after 3 days, 1 week, 15 days, 30 days, and 6-month intervals postoperatively. Specific observations were recorded like, pain (VAS scale), the size of the lesion, presence of any complications (ulceration, bleeding, infections, or scarring), and recurrences if any.

### 2.6. The Procedure of Sclerotherapy by 3% Sodium Tetradecyl Sulfate (STS) (Figure 4)

After administering the surface anesthesia with 15% xylocaine spray, each patient received an intralesional injection of 3% STS solution under the aseptic technique. After introducing the needle inside, the plunger was withdrawn to look out for the back-flow of blood if any, therefore, confirming accurate entry of the needle position into the center of the vascular space. The sclerosing agent was injected intralesionally at multiple sites into the mucosa: first, at the periphery, followed by the center of the lesion using an insulin syringe till the point of blanching was observed. Manual compression was applied for 15-20 minutes over the lesion to ensure hemostasis.

Larger lesions were treated by multiple injections. The usual dose of 3% sodium tetradecyl sulfate is 0.1 ml to 1.0 ml. The dose of the sclerosing agent usually depends on the size of the lesion (at a ratio of 0.2-0.3 ml for each 2 cm of lesion size, not exceeding 1 ml of the solution). This injection was repeated after an interval of 2 weeks again. Vascular lesions of the palate were not treated with sclerotherapy avoiding any chances of tissue necrosis and pain. Analgesics and anti-inflammatory drugs were prescribed to patients reported with signs/symptoms of inflammation. Similarly, in the laser group, the patients were reassessed after 3 days, 1 week, 15 days, 30 days, and 6 months postoperatively. VAS scale, the size of the lesion, presence of any complications (ulceration, edema, and ecchymosis), and recurrences (if any) were recorded.

The number of sessions for both laser treatment and sclerotherapy was dependent on certain factors such as (1) incomplete resolution of the lesion—based on the percentage of resolution of the size of the lesion, session varied; (2) size of the lesion—for voluminous lesion size more than 3.1 cm, laser treatment was based on the compartmentalization and serial step treatment as mentioned by Miyazaki et al. [15]; (3) lesser dose of the sclerosing agent in sclerotherapy—we preferred least possible dose of the sclerosing agent per session, hence the number of sessions increased.

## 3. Results

The study included a total of 40 patients (24 males and 16 females) presenting with oral vascular malformations (Table 1).

The patients were divided equally into two groups (20 in each), i.e., the laser group and sclerotherapy group. The patients in both the groups were matched on the site and size of the lesions. The difference between the site and size of the lesion between the two groups was statistically not significant with *p* values of 0.658 and 0.715, respectively (Table 2).

In the present study, vascular malformations were observed commonly in the tongue followed by buccal mucosa, lower labial mucosa, and upper labial mucosa. The number of treatment sessions was lesser in the laser group compared to the sclerotherapy group, but the comparison between the two was nonsignificant. The reason for more session in sclerotherapy group could be, firstly, more cases of a larger-sized lesion in the sclerotherapy group (5 cases of more than 3 cm sized lesion in sclerotherapy compared to 2 cases in laser group), and secondarily, we preferred lesser amount of injection per session as routine protocol. Side effects were found significantly lesser in the laser group compared to the sclerotherapy group (*p* value 0.044) (Table 2).

Postoperative pain (VAS scale) was compared between the two groups, and the difference was statistically significant (*p* value 0.04). The laser group had mild to moderate pain compared to severe pain in the sclerotherapy group. Comparison of response to treatment between the groups showed a nonsignificant difference (*p* value 0.376). Recurrence was observed more in the laser group compared to the sclerotherapy group (Table 2).

Site and size of involvement, detail of the number of sessions, side effects, pain (VAS scale), response of treatment, and recurrences are summarized in Table 2.

## 4. Discussion

Vascular malformations are limited to vessel structure without endothelial cell proliferation. These are formed by progressively enlarging aberrant and ecstatic vessels composed of the type of vessel involved, i.e., capillary, veins, and arteriovenous. Unlike hemangioma, vascular malformations are present at birth and rarely regress, and continue to expand, and have high rates of recurrence following the intervention [1–4]. Vascular malformations are mostly classified into two types: (a) based on the predominant type of blood vessel involved as lymphatic, capillary, venous, arteriovenous, and mixed malformations and (b) based on their hemodynamic flow characteristics as high-flow lesions (arterial malformations and arteriovenous malformations) and low-flow lesions (venous, capillary, and lymphatic malformations). Orofacial region commonly demonstrates low-flow vascular malformations (VMs) presenting on the lips and other areas of the oral mucosa like the ventral surface of the tongue and the buccal mucosa [1].

Various therapeutic modalities available for benign vascular diseases are sclerotherapy, embolization, surgical resection, cryotherapy, medical therapy (cortisone, beta-blockers), and laser treatment. The selection of the type of treatment modality depends on the type, location, and the depth and progression of the lesion [4, 5].

Laser therapy is among the mainstay in the management of vascular malformations. Few of the commonly used lasers include diode laser (800–980 nm), potassium–titanium–phosphate (KTP) laser (532 nm), neodymium–yttrium–aluminum–garnet (Nd:YAG) laser (1064 nm), pulsed dye laser (585 and 595 nm), argon laser (514 nm), and carbon dioxide (CO2) laser (10,600 nm) [6, 7].

The advantages of laser therapy are dry operating field, excellent visibility, minimum postoperative swelling, reduced postoperative pain, and less chance for mechanical trauma, therefore minimal scaring. Several other advantages are enhanced infection control along with the reduction of bacteria in blood, increased and better patient acceptance due to minimum postoperative discomfort, minimal requirement of local anesthesia for soft tissue treatments, minimal decontamination, and good bactericidal properties on the tissue which are observed. Therefore, there is reduced need for antibiotics postoperatively. [7, 8]

Disadvantages of laser are prolonged healing time required postoperatively due to the sealing of blood vessels and lymphatic in the surgical field and wounds take longer to reepithelialize. Lasers can cause ocular damage to the operator if an appropriate protective eyewear is not used. It is very important to be able to follow specific preventive measures strictly while performing the laser therapy. Laser systems are expensive and require additional training, leading to high cost of the procedure [7, 8].

The sclerotherapy procedure is one that involves the injection of a chemical solution (sclerosant) into a vein, which is known to damage the endothelial lining of the blood vessels, thereby causing vessel occlusion along with the development of fibrous tissue within. Variety of sclerosing agents have been tried clinically worldwide for several decades. Sodium tetradecyl sulfate sclerosant is one of the most commonly used agents owing to its efficacy. Each ml (milliliter) of 3% STS is known to contain 30 mg of sodium tetradecyl sulfate, 0.02 ml of benzyl alcohol, and 9.0 mg dibasic sodium phosphate; all mixed in water for injection at pH of 7.9. Monobasic sodium phosphate and/or sodium hydroxide are added for further pH adjustment, if required only [9, 10].

The dose of sclerotherapy injection was based on a study performed by Choi et al. They injected a minimum of 0.5–2 ml, STS 1% into each lesion, maximum of 2 ml. The volume was depending on a ratio of 0.5 ml for each 2 cm of lesion size or a quarter volume of the lesion. We have used lesser dose of 0.2-0.3 ml per 2 cm as the concentration of our solution was undiluted 3%. Thus, we suggest either low dose, high concentration or high dose, low concentration to avoid potential side effects [16].

Some of the advantages of sclerotherapy are its simple use, noninvasiveness, economical, safety, reliability, availability, and effective drug system with minimal side effects, known to cause minimal discomfort to the patient, nil or negligible blood loss, and no special requirement during postoperative dressings or any specific care [9, 10].

Sclerotherapy has few minor complications such as pain, sloughing, and ulceration, swelling, ecchymosis followed by tissue necrosis (Nicolau syndrome) and chances of anaphylaxis. Some of the major complications (only in large dermatological lesions) are pulmonary embolism, nerve damage, and disseminated intravascular coagulation [9, 10].

Many studies have been published regarding the use of laser [14–19] and sclerotherapy [20–24] for the treatment of vascular malformation/vascular lesions. Both of the techniques are proven to be effective to decrease the size of the lesion significantly. Laser has also been used for the surgical excision of small vascular malformations [25]. However there is no study published comparing these two efficient treatment modalities of oral vascular malformation.

Our study showed that both the techniques were significantly effective in the treatment of vascular malformation. On comparing the two, a diode laser was found better compared to sclerotherapy considering the adverse reactions like postoperative pain and edema. Sclerotherapy group had lesser recurrences than laser group. The study group of five patients—laser group shows recurrences compared to one recurrence in sclerotherapy group. This showed that the sclerosing agents act deeper and irreversibly compared to the laser group. Most of the recurrences were associated with the larger-sized lesion. Thus, our study concluded that both the techniques were effective. Laser was better for smaller lesion, and sclerotherapy was better for the larger lesion.

Imaging plays an important role in the diagnosis of vascular malformations. Ultrasound specifically color Doppler and MRI can identify high-flow patterns as well as determine the extent of the lesion. Magnetic resonance imaging is especially useful in defining the extent of vascular malformation. Computerized tomography (CT) can be valuable, especially for bony vascular malformations. Angiography can also be utilized for defining the feeding and draining vessels prior to sclerotherapy or surgical intervention [26]. Doppler ultrasound and CT angiography were performed in all our cases.

### 4.1. Limitations of the Study

The data was collected by the same authors who administered the treatment in their respective departments. Postoperative imaging assessment should be encouraged. However, due to financial constraints and limited resources, we were unable to carry out these in all the subjects.

## 5. Conclusion

Laser and sclerotherapy with 3% sodium tetradecyl sulfate both are effective in the treatment of vascular malformations. These techniques offer an effective alternative to traditional or conventional methods like surgery, radiofrequency ablation, electrodesiccation procedures with success, and less chance of recurrences. Diode laser seems to be better than sclerotherapy given lesser side effects and patient comfort. For small lesions or those located in the areas where esthetically conservation is required and the lesions where chances of necrosis are high like the palate, the laser is considered to be better (due to lesser adverse effect) while for larger lesions sclerotherapy can be considered.

## Figures and Tables

**Figure 3:**
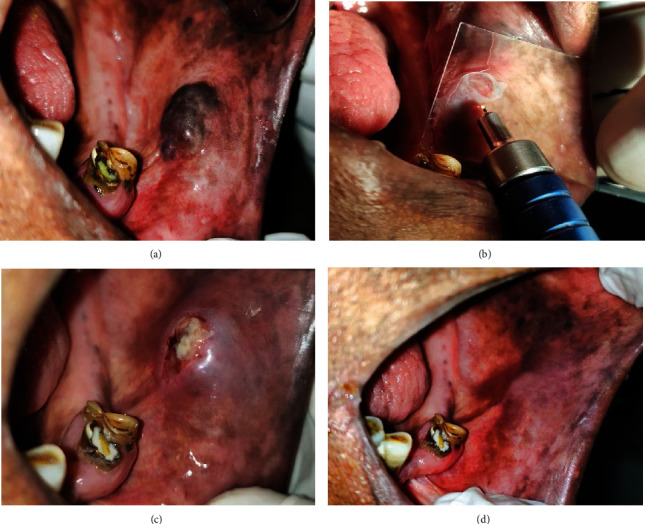
Treatment of a vascular malformation with diode laser by photocoagulation of the lesion (noncontact mode, 2 watt, 980 nm Indilase). (a) Presence of vascular malformation. (b) Laser exposure is done in noncontact mode 2-3 mm away from the lesion. We can use glass slide to avoid accidental exposure Just after laser procedure, presence of blanching is the indicator of photocoagulation. (c) After 3 days, ulcer is formed in place of vascular malformation. (d) Healing completed with complete disappearance of lesion.

**Figure 4:**
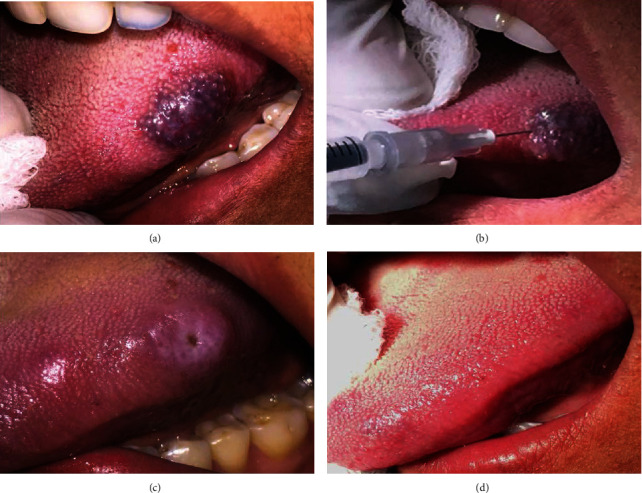
Treatment of a vascular malformation with sclerotherapy (sodium tetradecyl sulfate). (a) Presence of vascular malformation. (b) Intralesional injection of 0.2 ml sodium tetradecyl sulfate 3%. (c) Appearance of an ulcer after 3-4 days of intralesional injection. (d) Complete disappearance of the lesion.

**Table 1:** Details of gender.

Gender	Frequency	Percent
Male	24	60
Female	16	40
Total	40	100

**Table 2:** Details of comparison between diode laser and sclerotherapy.

Details of site of involvement			
	Laser group	STS group	Chi sq 2.424 *p* value 0.658^NS^
Tongue	9 (45%)	9 (45%)	
Buccal mucosa	5 (25%)	6 (30%)	
Lower labial mucosa	3 (15%)	3 (15%)	
Upper labial mucosa	1 (5%)	2 (10%)	
Palate	2 (10%)	0	
Total	20 (100%)	20 (100%)	

Details of size of involvement			
Less than 1 × 1 cm	8 (40%)	7 (35%)	Chi sq 0.722 *p* value 0.715^NS^
1.1 × 1.1 cm-2.0 × 2.0 cm	6 (30%)	4 (20%)	
2.1 × 2.1 cm-3.0 × 3.0 cm	4 (20%)	4 (20%)	
More than 3.1 cm	2 (10%)	5 (25%)	
Total	20(100%)	20 (100%)	

Details of the number of session			
1 session	10 (50%)	12 (60%)	Chi sq 1.143 *p* value 0.565^NS^
2 session	7 (35%)	4 (20%)	
3 or more than 3 session	3 (15%)	4 (20%)	
Total	20 (100%)	20 (100%)	

Details of the side effects			
Pain	15 (75%)	20 (100%)	Chi sq 4.051 *p* value 0.044^∗^
Edema	0	20 (100%)	
Superficial ulceration	20 (100%)	20 (100%)	
Mild ecchymosis	0	1 (5%)	

Details of the VAS scale on 3^rd^ postoperative day			
7-10 VAS	0	14 (70%)	Chi sq 29.091 *p* value < 0.001^∗∗^
4-6 VAS	5 (25%)	6 (30%)	
1-4 VAS	10 (50%)	0	
0 VAS/no pain	5 (25%)	0	
Total	20 (100%)	20 (100%)	

Response of treatment to individual groups			
Complete response	16 (80%)	18 (90%)	Chi sq 0.784 *p* value 0.376^NS^
Moderate response	4 (20%)	2 (10%)	
No response	0	0	
Total	20 (100%)	20 (100%)	

Details of the recurrences			
Recurrences	5 (25%)	1 (5%)	—
Total	20 (100%)	20 (100%)	

## Data Availability

The data supporting the results of this study were obtained from the Department of Oral Medicine and Radiology, Nobel Medical College and Teaching Hospital, Biratnagar, Nepal. The data used are included within the article and are also available by email to the corresponding author.
